# Endoscopic endonasal approach for mass resection of the pterygopalatine fossa

**DOI:** 10.6061/clinics/2017(09)06

**Published:** 2017-09

**Authors:** Jan Plzák, Vít Kratochvil, Adam Kešner, Pavol Šurda, Aleš Vlasák, Eduard Zvěřina

**Affiliations:** I Department of Otorhinolaryngology and Head and Neck Surgery, 1^st^ Faculty of Medicine, Charles University, University Hospital Motol, V Úvalu 84, 150 06, Prague 5, Czech Republic; II Guy's and St Thomas' NHS Foundation Trust, Great Maze Pond, SE1 9RT London, UK; III Department of Neurosurgery, 2^nd^ Faculty of Medicine, Charles University, University Hospital Motol, V Úvalu 84, 150 06, Prague 5, Czech Republic

**Keywords:** Endoscopic Endonasal Approach, Pterygopalatine Fossa, Skull Base, Tumor

## Abstract

**OBJECTIVES:**

Access to the pterygopalatine fossa is very difficult due to its complex anatomy. Therefore, an open approach is traditionally used, but morbidity is unavoidable. To overcome this problem, an endoscopic endonasal approach was developed as a minimally invasive procedure. The surgical aim of the present study was to evaluate the utility of the endoscopic endonasal approach for the management of both benign and malignant tumors of the pterygopalatine fossa.

**METHOD:**

We report our experience with the endoscopic endonasal approach for the management of both benign and malignant tumors and summarize recent recommendations. A total of 13 patients underwent surgery via the endoscopic endonasal approach for pterygopalatine fossa masses from 2014 to 2016. This case group consisted of 12 benign tumors (10 juvenile nasopharyngeal angiofibromas and two schwannomas) and one malignant tumor.

**RESULTS:**

No recurrent tumor developed during the follow-up period. One residual tumor (juvenile nasopharyngeal angiofibroma) that remained in the cavernous sinus was stable. There were no significant complications. Typical sequelae included hypesthesia of the maxillary nerve, trismus, and dry eye syndrome.

**CONCLUSION:**

The low frequency of complications together with the high efficacy of resection support the use of the endoscopic endonasal approach as a feasible, safe, and beneficial technique for the management of masses in the pterygopalatine fossa.

## INTRODUCTION

**Figure 1:**
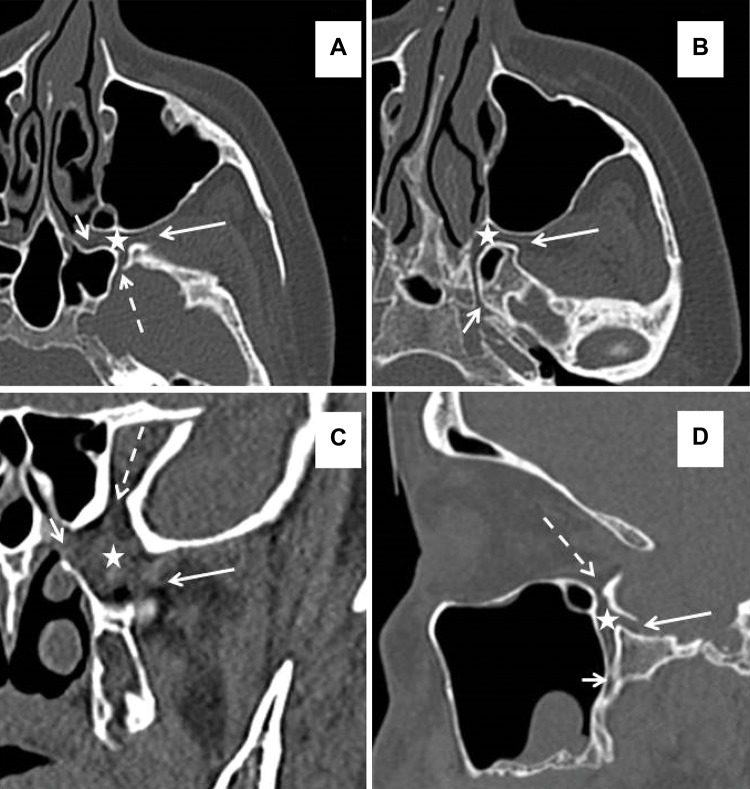
CT anatomy of the pterygopalatine fossa (*asterisk*). (A) An axial image showing a communication to the ITF via the pterygomaxillary fissure *(long arrow*) to the middle cranial fossa via the foramen rotundum *(dashed arrow)* and to the nasal cavity via the sphenopalatine foramen *(short arrow)*. (B) An axial image in a different plane showing the Vidian canal running to the foramen lacerum *(short arrow)*. (C) A coronal image showing a communication to the nasal cavity via the sphenopalatine foramen *(short arrow)* to the orbit via the infraorbital fissure *(dashed arrow)* and to the ITF via the pterygomaxillary fissure *(long arrow*). (D) A sagittal image showing the greater palatine canal *(short arrow)* running to the oral cavity with a communication to the orbit via the infraorbital fissure *(dashed arrow)* and to the middle cranial fossa via the foramen rotundum *(long arrow)*.

The pterygopalatine fossa (PPF) is a narrow, inverted, cone-shaped space that is localized posteriorly to the dorsal wall of the maxillary sinus and consists of fat, the pterygopalatine ganglion, the Vidian nerve, the maxillary nerve (V2), and terminal branches of the maxillary artery. The anteroposterior order of the layers is as follows: fat, blood vessels, and neural structures. The posterior wall of the PPF is formed by the base of the pterygoid process and is bordered by the palatine bone medially and the middle cranial fossa cranially. Laterally, the PPF is open to the infratemporal fossa (ITF) via the pterygomaxillary fissure. The Vidian canal opens into the posterior part of the PPF and contains the Vidian nerve and artery. The palatovaginal (pharyngeal) canal is located medially to the Vidian canal, and the sphenopalatine foramen is a communication medial to the nasal cavity via the palatine bone. The descending palatine canal continues inferiorly to the apex of the PPT and opens into the oral cavity via the greater and lesser palatine foramina. The foramen rotundum, containing V2, is a communication that is located cranially to the middle crania fossa. The inferior orbital fissure is open anterosuperiorly to the orbit. The PPF is a very complex anatomical area of the skull base with interconnections to the head and neck (Figure 1) 1–3.

Accessing the PPF is difficult, which is traditionally approached via an open method, such as lateral rhinotomy, midfacial degloving, facial translocation, transantral maxillectomy, and the Fisch C and D procedures. Although these procedures provide good exposure of the PPF, they are often complicated by unacceptable facial scaring and deformity as well as dysfunction of the facial and infraorbital nerves. Endoscopic sinus surgery is now a standard procedure for inflammatory sinonasal disease, and more recently, it has been adopted as a basic approach for the treatment of benign sinonasal tumors, such as inverted papilloma or juvenile nasopharyngeal angiofibroma (JNA) 4,5. Advances in endoscopic endonasal surgery, such as interventional radiology with preoperative embolization, improved instrumentation, computer-based navigation, and hemostatic materials, as well as a two-surgeon technique, have enabled endoscopic endonasal access to tumors of the PPF 6. Primary tumors of the PPF, such as schwannomas of V2, are rare, while sinonasal tumors more frequently extend into the PPF 7. Hence, relatively few studies (most of which were case series involving low numbers of patients) have investigated the endoscopic endonasal approach (EEA) to access tumors of the PPF 8–11. Our study follows the recommendations of previous authors, who called for the publication of more cases. Therefore, the surgical aim of the present study was to evaluate the utility of the EEA for the management of both benign and malignant tumors of the PPF. The present study included the highest number of cases per year among related published reports. Adequate follow-up and appropriate postoperative care (a novelty of trismus physiotherapy) were emphasized.

## MATERIAL AND METHODS

The surgical records of 13 patients who underwent resection of a tumor of the PPF via the EEA at the Department of Otorhinolaryngology and Head and Neck Surgery, 1^st^ Faculty of Medicine, Charles University, University Hospital Motol, Prague, Czech Republic from April 2014 to March 2016 were retrospectively reviewed. All patients who underwent resection via the EEA for a tumor of the PPF during this period were enrolled. The study protocol was approved by the institutional review board of our hospital, and written informed consent was obtained from all patients.

Diagnosis was based on patient history and the results of complete clinical otorhinolaryngological examinations, which included nasal endoscopy, computed tomography (CT), and magnetic resonance imaging (MRI). No biopsy was performed for 10 patients with clinical and imaging findings that were consistent with a diagnosis of JNA. For confirmed cases of JNA, preoperative angiography with embolization was performed one day before surgery. Two patients had schwannomas of V2: one was biopsied from a portion of the tumor in the lateral part of the choana at an otorhinolaryngology department in another hospital, and the second was diagnosed with a high probability based on preoperative assessment and was subsequently confirmed by perioperative biopsy. The last patient in this cohort was the only case with malignancy. In this patient, undifferentiated sinonasal carcinoma (cT4b cN0) infiltrated the bilateral nasal cavities, bilateral ethmoids, unilateral maxillary and sphenoid sinuses, unilateral PPF, and the brain in the anterior cranial fossa. This was the only case to undergo combination surgery via the EEA for the management of the sinonasal and pterygopalatine portions of the tumor and bifrontal craniotomy for the intracranial portion. This patient also received adjuvant chemoradiotherapy. Postoperative follow-up of all cases included clinical examination with an emphasis on endoscopic evaluation and radiological workup according to European guidelines 4.

All surgeries were performed under general anesthesia. In 11 cases of sinonasal tumors extending to the PPF (10 cases of JNA and one case of undifferentiated sinonasal carcinoma), the tumor in the nasal cavity blocking the approach to the posterior wall of the maxillary sinus was resected using a piecemeal technique to expose the surgical intranasal corridor as the first step. The nasal septum was resected in cases of undifferentiated sinonasal carcinomas due to tumor infiltration and in schwannoma cases with large extensions to the middle cranial fossa, ITF, and cheek to safely achieve a larger working space. The second step (the ethmoid step) involved anteroposterior ethmoidectomy, sphenoidectomy, extensive supraturbinal antrostomy, and partial resection of the middle (dorsal part) and superior turbinates. Antrostomy was extended to the posterior wall of the maxillary sinus. In cases of infiltration of undifferentiated sinonasal carcinoma, complete medial maxillectomy was performed. Complete medial maxillectomy was also performed in cases of giant schwannoma (P11) extending from the PPF through the ITF laterally from the sagittal plane passing at the lateral border of the maxillary sinus to ensure sufficient lateral access. The sphenopalatine foramen and sphenopalatine artery entering the nasal cavity near the tip of the middle turbinate was identified as an important surgical landmark. The posterior wall of the maxillary sinus was then removed while preserving the periosteum of the PPF as the third step. Preservation of the periosteum surfacing the PPF was helpful for subsequent identification of the Vidian nerve medially and the maxillary nerve superolaterally. The extent of the surgical window in the posterior wall was dependent on the exact location and extent of the tumor in the PPF. The extent of drilling around the sphenopalatine foramen was also determined based on the extent of PPF involvement. The fourth step was appropriate resection of the PPF tumor. The first structure encountered after incision of the PPF periosteum was fat tissue, followed by blood vessels. The distal branches of the maxillary artery (Vidian, descending palatal, and palatovaginal branches) and its main trunk were identified and cauterized. The neural structures all lie deeper than this plexus of arteries. During blunt dissection of the tumor, maximal effort was taken to preserve the maxillary and infraorbital nerves, the greater and lesser palatine nerves, and the Vidian nerve (if they were not involved in the tumor) to minimize neurological morbidity. The posterolateral border of dissection was the anterior surface of the lateral pterygoid muscle. Navigation with CT/MRI fusion and perioperative frozen sections were used to ensure safe radical resection. The final step consisted of drilling around the Vidian canal to prevent JNA recurrence. In case of malignancy, the pterygoid root and plates were also resected.

## RESULTS

**Figure 2:**
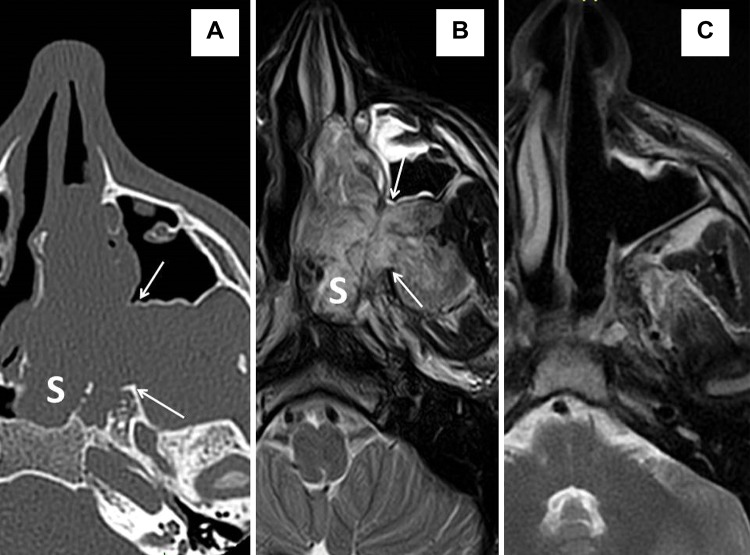
Juvenile nasopharyngeal angiofibroma, case P5, Radkowski grade 2c. Axial CT (A) and MRI T2-weighted scans (B) showing a mass in the dorsal two thirds of the left nasal cavity and in the sphenoid sinus *(marked by S)* with a bulky extension to the pterygopalatine fossa and the ITF. Extensive anteroposterior widening of the sphenopalatine foramen is marked by *arrows*. (C) An axial MRI T2-weighted scan one year after surgery demonstrating no evidence of disease.

**Figure 3:**
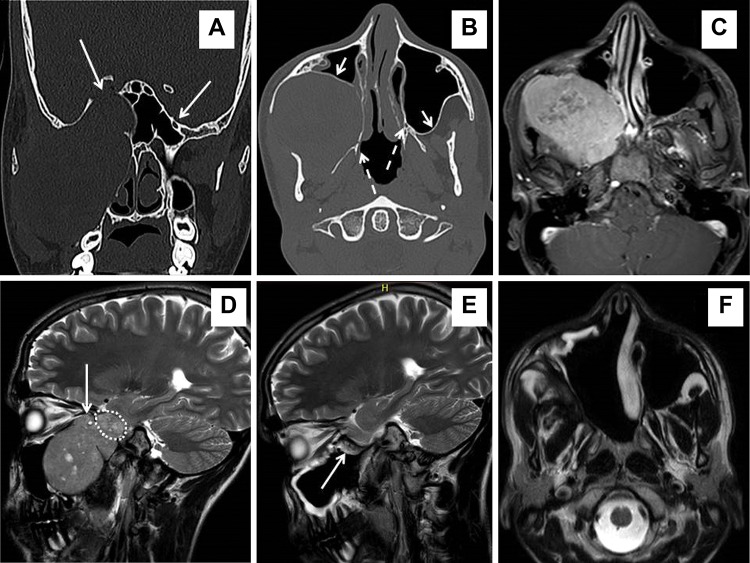
Schwannoma of V2, case P11. Coronal (A) and axial CT (B) scans showing considerable deformation of the splanchno and neurocranium on the right side. A mass (74 x 65 x 61 mm) arising from V2 in the pterygopalatine fossa with bulky extension to the ITF, cheek, and middle cranial fossa via the foramen rotundum, which is extremely enlarged *(long arrow)* – see side difference. The schwannoma pushed the posterior wall of the maxillary sinus forward *(short arrow)* and the pterygoid process backward *(dashed arrow)* – also see side differences. (C) An axial T1-weighted MRI scan with gadolinium contrast showing the margin between the tumor and soft tissue. (D) A sagittal T2-weighted MRI scan showing that the tumor is pressing on the orbital apex via the infraorbital fissure *(long arrow)* with an intracranial portion (24 x 21 x 20 mm) in the middle cranial fossa *(dotted line)*. Postoperative T2-weighted MRI scans in the sagittal (E) and axial (F) planes demonstrate no evidence of disease 21 months after surgery. A nasoseptal flap *(long arrow)* was used to cover exposed dura mater of the middle cranial fossa, although no clear leakage of cerebrospinal fluid was observed intraoperatively. Considerable retraction of soft tissue into the postresection empty space of the cheek and ITF is visible on the axial MRI – compare (C) and (F).

**Figure 4:**
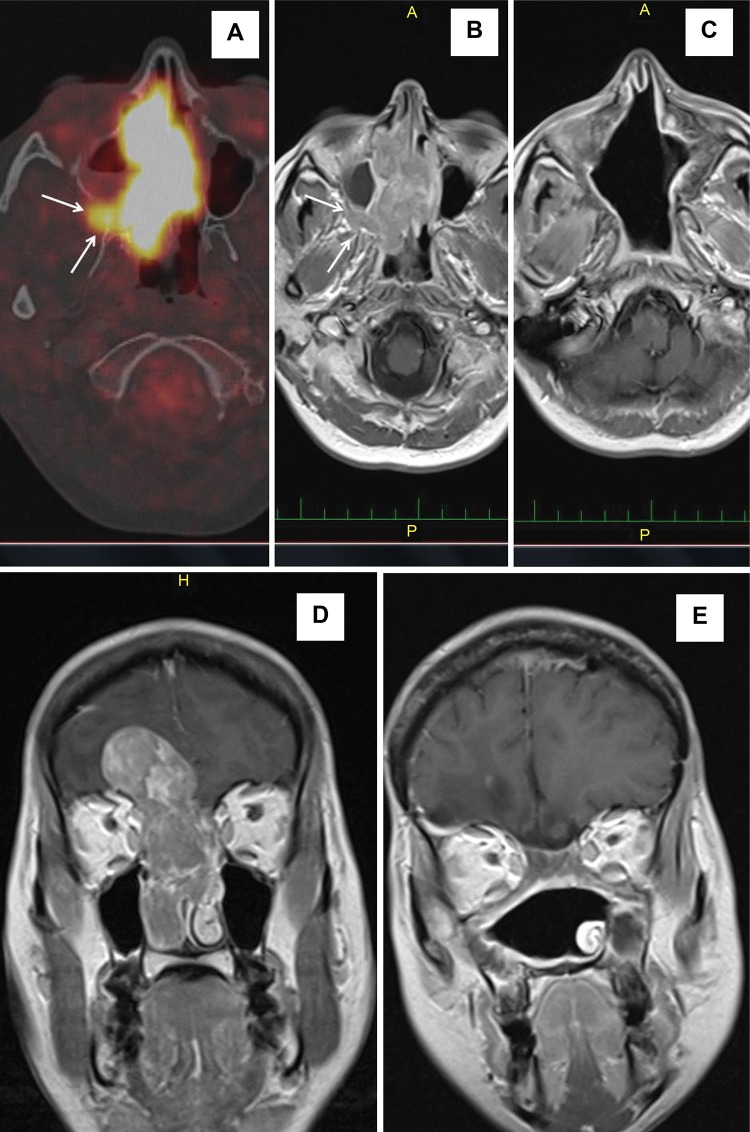
Sinonasal undifferentiated carcinoma T4b, case P13. Axial PET/CT (A) and axial (B) and coronal (D) MRI T1-weighted with gadolinium contrast images showing a mass in the bilateral nasal cavities, bilateral ethmoid sinuses, right maxillary and sphenoid sinuses, and right pterygopalatine fossa *(arrows)* with an intracranial portion with brain infiltration. Axial (C) and coronal (E) MRI T1-weighted scans with gadolinium contrast demonstrating no evidence of disease two years after surgery and postoperative chemoradiotherapy.

The group of 13 patients with tumors of the PPF who were treated via the EEA included 11 males and 2 females with a mean age of 21.8 (range, 15–56) years (Table 1). The following symptoms were observed among these 13 patients: unilateral nasal obstruction in 12 patients, epistaxis in six patients, and pain in four patients. One patient (P12) with a smaller schwannoma had no clinical symptoms with incidental finding on MRI. A total of 12 patients had benign tumors: 10 cases of JNA (3x IIa, 1x IIb, 5x IIc, and 1x IIIb according to the Radkowski grading system, i.e., six cases with extension of the tumor out of the PPF to the ITF – grade IIc and/or intracranially – grade IIIb) and two with schwannomas arising from the maxillary nerve. Representative cases of JNA (P5) and schwannoma (P11) are shown in Figures 2 and 3, respectively. One malignant tumor was undifferentiated sinonasal carcinoma (cT4b cN0) that infiltrated the bilateral nasal cavities, bilateral ethmoids, unilateral maxillary and sphenoid sinuses, and unilateral PPF, including the intracranial portion with brain infiltration (Figure 4). This patient was the only one that underwent combination surgery, i.e., EEA for the management of the sinonasal and pterygopalatine portions of the tumor and bifrontal craniotomy for the intracranial portion. This patient was also the only one to receive adjuvant chemoradiotherapy using proton beam therapy. The optimal management of sinonasal undifferentiated carcinoma remains unclear. Treatment strategies vary among institutions, and no single approach has demonstrated a clear therapeutic advantage. Surgery as the primary mode of treatment with postoperative chemoradiotherapy is acceptable for patients with resectable disease and improves oncologic control 4,13. Ten tumors (all JNAs) originated in the nasopharynx with extension to the PPF (in six cases, more laterally to the ITF). Both schwannomas of V2 were primary tumors of the PPF. Seven patients with JNA in this cohort underwent primary surgery, and three were revision surgeries. Case P2 underwent external lateral rhinotomy 2 years before endoscopic revision for a growing residual tumor in the PPF and ITF. Case P7 was a giant JNA that was endoscopically resected three times in 2 years at another otorhinolaryngology department. The last surgery was performed without preoperative embolization due to the blood supply from the internal carotid artery. This surgery was stopped prematurely due to uncontrollable bleeding from the tumor. Subsequent multiple attempts for nasal tamponade removal were unsuccessful due to continued uncontrollable bleeding, and the patient was transported to the Department of Otorhinolaryngology and Head and Neck Surgery, 1^st^ Faculty of Medicine, Charles University with a residual JNA in the PPF and ITF. Subsequent R0 endoscopic resection was performed without preoperative embolization. JNA case P8 underwent endoscopic resection twice in 2 years at another otorhinolaryngology department. A residual tumor remained in the PPF and ITF; therefore, the patient underwent revision surgery at the Department of Otorhinolaryngology and Head and Neck Surgery, 1^st^ Faculty of Medicine, Charles University.

Of the 13 patients, 12 had no microscopic residual disease (R0). In case of JNA P6 (stadium Radkowski grade 3b – intracranial extension with cavernous sinus invasion), a residual tumor of approximately 1 cm in diameter was left in the cavernous sinus area. Repetitive postoperative MRI at 6-month intervals showed that the residual tumor was stable. No development of a new residual or recurrent tumor was observed in any of the other cases during the follow-up period.

The mean blood loss during surgery was 550 (range, 100–1,700) mL. Ten patients received intraoperative transfusions. No significant intraoperative neuro/vascular complications occurred. The mean duration of postoperative hospitalization was 5.8 (range, 4–12) days. There were no postoperative infections or systemic complications. Postoperative neural dysfunction was observed in eight cases: four cases of transient V2 hypesthesia and four cases of permanent V2 hypesthesia (two cases of V2 schwannoma; JNA Radkowski grade 3b in case P6 and JNA Radkowski grade 2c after three previous non-radical surgeries in case P7). Dry eye syndrome due to pterygopalatine ganglion dysfunction occurred in one case. Permanent trismus was present in two cases: one with a JNA (case P7) and one with a carcinoma (case P13), in which deterioration mainly occurred after chemoradiotherapy.

## DISCUSSION

The management of PPF lesions represents one of the areas in which open surgery has been shifted back to the stage of the last decade. Endoscopic endonasal surgery is performed for lesions with boundaries that extend beyond the paranasal cavities to the PPF and ITF 12 and is indicated not only for inflammatory disease but also for benign and even malignant tumors that are very difficult to reach in this anatomical region 13. The use of a natural pathway via the nasal and paranasal cavities reduces morbidity associated with the access route compared to the external approach. Cosmesis, especially external scarring, is also an important factor in favor of the EEA. Angled endoscopes provide much better visualization of the PPF, which is difficult to access. A profound knowledge of the PPF and ITF and their neighboring spaces is essential for performing safe surgery. The creation of an appropriate endoscopic surgical corridor is crucial to allow safe manipulation, tissue dissection, and hemostasis. It is more difficult to manage minor bleeding using the EEA than when using an open approach. In addition, a narrower variety of instruments are usable for mass dissection in the EEA.

Biopsy as well as radical resection of PPF tumors via the EEA have been reported in the literature 14-16. The case series reported by Battaglia et al. 11 describing 37 patients who were treated during a 13-year period is among the most extensive. Our study follows the recommendations of this group, which called for the publication of more cases. The present study included the highest number of cases per year among related published reports.

The PPF is one of the most anatomically complex regions of the human body because it contains a range of vital neural and vascular structures with interconnections to the orbit, middle cranial fossa, nasal cavity, and oral cavity 17,18. All of these structures must be carefully considered when planning surgery and especially during the surgical procedure. Preservation of the neural structures in the PPF is necessary to limit surgical morbidity. Vascular complications are mostly caused by damage to branches of the maxillary artery. The management of massive blood loss is one of the limiting steps of the EEA to access the PPF.

No major intraoperative complications occurred among the 13 patients in this cohort, while 9 (69.2%) developed minor complications, which included transient complications in three cases (V2 hypesthesia and trismus). Permanent postoperative sequelae were present in 6 (46.1%) patients, which included dry eye syndrome in one case, V2 hypesthesia in three cases, V2 hypesthesia with trismus in one case, and trismus in one case. V2 damage was associated with extensive PPF involvement in advanced JNA and with schwannomas arising from V2. Resection extending to the pterygoid muscle is thought to induce postoperative trismus. Therefore, we recommend trismus physiotherapy for all PPF surgeries. Despite this precaution, permanent trismus was observed in two cases. Case P7 developed slight permanent trismus after surgery for JNA, which might have resulted from the three previous non-radical surgeries. One case of carcinoma (case P13) developed permanent trismus after postoperative chemoradiotherapy. In case P11 with giant trigeminal schwannoma originating from the pterygopalatine fossa, we succeeded with the treatment of postoperative trismus by physiotherapy, and the patient is now free of this symptom. All other patients did not develop trismus, presumably because of early rehabilitation of mouth opening starting on the 2^nd^ to 5^th^ postoperative days. The patients were instructed during self-rehabilitation to perform a daily exercise of mouth opening.

The use of total endoscopic endonasal management of residual/recurrent disease located in the PPF remains controversial. There were three such cases in our study, all of which were JNAs. Cases P7 and P8 were treated first by repeated endonasal endoscopy via the EEA at other departments. After careful evaluation of images, we found no resections of the posterior wall of the maxillary sinus. Therefore, we expected no previous manipulation in the PPF and no scarring. Our presumptions were confirmed during the surgeries; thus, radical endonasal endoscopic resections were performed. The third patient (P2) was the last who underwent external surgery for JNA at our Department of Otorhinolaryngology. Additionally, in this case, there was no intervention in the PPF through the posterior wall of the maxillary sinus during the primary external surgery; thus, subsequent revision endoscopic surgery for a growing mass in the PPF was successful.

The low frequency of complications together with the high efficacy of resection (no recurrence and only one stable persistent residuum in the cavernous sinus) support endoscopic endonasal surgery as a feasible, safe, and beneficial technique for the management of masses in the PPF. The following are fundamental prerequisites to safely and effectively performing this approach: 1 thorough preoperative evaluation of imaging examinations (MRI/CT), 2 the availability of adequate technical equipment (endoscopes, endoscopic skull base instruments, a navigation system, hemostatic facilities, etc.), and 3 adequate experience and skill of the surgeon in the use of advanced endoscopic techniques.

## AUTHOR CONTRIBUTIONS

Plzák J and Zvěřina E were responsible for the study design, surgeons, manuscript writing and supervision of the study. Kratochvil V, Kešner A, Šurda P and Vlasák A were responsible for the data collection and processing, surgeons, and manuscript revision

## Figures and Tables

**Table 1:** Summary of the clinical characteristics of patients undergoing an endonasal endoscopic approach to the pterygopalatine fossa.

Case	Age (yr)	Sex	Diagnosis	Origin	Extension	Maximal laterolateral size in PPF /+ITF/(mm)	Previous treatment	Preoperative management	Resection	Adjuvant therapy	Follow-up (mo)	Sequelae
P1	18	M	JNA Radkowski 2c	NP	PPF, ITF	32	0	embolization	R0	0	16	transient V2 hypesthesia
P2	18	M	JNA Radkowski 2c	NP	PPF, ITF	29	lat. rhinotomy 2 yrs ago	embolization	R0	0	17	transient V2 hypesthesia
P3	15	M	JNA Radkowski 2a	NP	PPF	9	0	embolization	R0	0	25	
P4	16	M	JNA Radkowski 2a	NP	PPF	11	0	embolization	R0	0	28	
P5	16	M	JNA Radkowski 2c	NP	PPF, ITF	36	0	embolization	R0	0	29	dry eye syndrome, transient V2 hypesthesia
P6	16	M	JNA Radkowski 3b	NP	PPF, ITF, MCF	34	0	embolization	R1 (cavernous sinus)	0	29	V2 hypesthesia
P7	15	M	JNA Radkowski 2c	NP	PPF, ITF	16	3x endoresection at another department	0 (ICA supply)	R0	0	30	V2 hypesthesia, trismus
P8	15	M	JNA Radkowski 2c	NP	PPF, ITF	33	2x endoresection at another department	embolization	R0	0	30	transient V2 hypesthesia
P9	16	M	JNA Radkowski 2b	NP	PPF	19	0	embolization	R0	0	37	
P10	24	M	JNA Radkowski 2a	NP	PPF	7	0	embolization	R0	0	39	
P11	15	M	Schwannoma	PPF	PPF, ITF, cheek, MCF	61	0	0	R0	0	34	V2 hypesthesia, transient trismus
P12	56	F	Schwannoma	PPF	PPF, ITF	32	0	0	R0	0	19	V2 hypesthesia
P13	44	F	SNUC T4bN0 M0	SN	PPF	21	0	0	R0	CHRT (proton beam therapy)	36	trismus

Abbreviations: M = male; F = female; JNA = juvenile nasopharyngeal angiofibroma; SNUC = sinonasal undifferentiated carcinoma; NP = nasopharynx; PPF = pterygopalatine fossa; SN = sinonasal; ITF = infratemporal fossa; MCF = middle cranial fossa; ICA = internal carotid artery; CHRT = chemoradiotherapy
